# Identification and characterization of single nucleotide polymorphism markers in *FADS2* gene associated with olive oil fatty acids composition

**DOI:** 10.1186/s12944-017-0530-6

**Published:** 2017-07-12

**Authors:** Rayda Ben Ayed, Karim Ennouri, Hajer Ben Hlima, Slim Smaoui, Mohsen Hanana, Rim Mzid, Sezai Ercişli, Ahmed Rebai

**Affiliations:** 1 Molecular and Cellular Screening Processes: Research group, Microorganisms and Biomolecules Laboratory, Centre of Biotechnology of Sfax, BP ‘1177’ Sidi Mansour road, 3018 Sfax, Tunisia; 2 Unité de Biotechnologie des Algues, Biological Engineering Department, National School of Engineers of Sfax, University of Sfax, Sfax, Tunisia; 3 Laboratory of Microorganisms and Biomolecules, Center of Biotechnology of Sfax, PB 1177, 3018 Sfax, Tunisia; 4 Laboratory of Extrêmophile Plants, Biotechnology Center of Borj-Cédria, B.P. 901, 2050 Hammam Lif, Tunisie; 5 Ataturk University, Faculty of Agriculture, Department of Horticulture, 25240 Erzurum, Turkey

**Keywords:** Olive oil, SNP genotype, Quality, Fatty acid composition, Oleic acid, *FADS2*

## Abstract

**Background:**

Genotyping of the FAD2.1 and FAD2.3 polymorphisms in the *fatty acid desaturase 2* gene (*FADS2*) shows that they are associated with the fatty acids composition of olive oil samples. However, these associations require further confirmation in the Tunisian olive oil cultivars, and little is known about the effect of polymorphisms in fatty acid-related genes on olive oil mono- and poly- unsaturated fatty acids distribution.

**Methods:**

A set of olive oils from 12 Tunisian cultivars was chosen. The fatty acid composition of each olive oil sample was determined by gas chromatography. Statistical and modeling Bayesian analyses were used to assess whether the FAD2.1 and FAD2.3 genotypes were associated with fatty acids composition.

**Results:**

The TT-FAD2.1 and the GG-FAD2.3 genotypes were found to be associated with a lower proportion of oleic acid (C18:1) (*r* = −0.778, *p* = 0.003; *r* = −0.781, *p*= 0.003) as well as higher proportion of linoleic (C18:2) (*r* = 0.693, *p* = 0.012; *r* = −0.759, *p*= 0.004) and palmitic acids (C16:0) (*r* = 0.643, *p* = 0.024; *r* = −0.503, *p*= 0.095), making varieties with this haplotype (i.e. Chemlali Sfax and Meski) producing more saturated (C16: 0) and polyunsaturated acids than oleic acid. The latter plays a major role in preventing several diseases.

**Conclusion:**

The two associations *FADS2* FAD2.1 and *FADS2* FAD2.3 with the fatty acid compositions of olive oil samples were identified among the studied olive cultivars. These associations differed between studied cultivars, which might explain variability in lipidic composition among them and consequently reflecting genetic diversity through differences in gene expression and biochemical pathways. *FADS2* locus would constitute thus a good marker for detecting interesting lipidic chemotypes among commercial olive oils.

## Background

Olive (*Olea europaea* L.) is one of the oldest agricultural plants and is an important oil-producing crop in the Mediterranean Basin. The oil obtained from its drupes can be consumed in crude form and is known for its nutritional and healthy features compared to other vegetable oils [1, 2].

The major compounds in olive oil are glycerides corresponding to more than 98% of the total weight. Oleic acid (C18: 1) is a monounsaturated fatty acid corresponding to 55–85% of total fatty acids, while the most essential polyunsaturated fatty acid in our diet, the linoleic acid (C18: 2), represents 3 to 21% [3].

Many studies have described the beneficial effects of olive oil consumption on health and especially on blood pressure [4]. These studies highlighted the close relationship between blood pressure and the total fat intake as well as saturated-unsaturated fat ratio. Recent studies have pointed out the possibility that olive oil possesses an antihypertensive effect because of its high content of oleic acid [5].

The goal of the present paper is to investigate the association between SNP (Single Nucleotide Polymorphism) and olive oil quality parameters and to evaluate their possible usefulness in olive oil authenticity. Indeed, the work highlights two SNPs localized in the *FADS2 (fatty acid desaturase 2)* gene namely FAD2.1 and FAD2.3. The *FADS2* gene is involved in the biosynthesis of highly unsaturated fatty acids from polyunsaturated fatty acids precursor [6]. Particularly, this study aims to evaluate these SNPs and their association with fatty acids composition and to identify SNPs usefulness in the chemotype authentication and characterization of Tunisian olive oils.

## Methods

### Plant material

A total of twelve Tunisian olive-tree cultivars were chosen from different geographical regions of Tunisia from north to south (Chetoui, Tounsi, Meski, Oueslati, El Horr, Chemlali Sfax, Chemlali Tataouine, Zarrazi, Chemchali, Besbessi, Fougi and Toffehi). For each cultivar, two trees were used for leaf sampling.

### DNA isolation

The DNA was extracted from leaves using the CTAB method described by Ben Ayed et al. [7] and an additional purification was introduced, consisting in washing and eluting once with the QIAamp DNA stool (Qiagen) to eliminate contaminant molecules and generate a high quality DNA for specific, reproducible and consistent amplifications [7]. Genomic DNA was dissolved in TE buffer (10 mM Tris–HCl pH 8.1 mM EDTA pH 8) and stored at −20 °C.

### SNP genotyping

Two SNPs were selected within the *FADS2* locus which is involved in the biosynthesis of highly unsaturated fatty acid. These two SNPs (FAD2.1 and FAD2.3) were genotyped by a polymerase chain reaction-restriction fragment length polymorphism (PCR-RFLP) method. The PCR products (241 and 240 bp) of the SNPs (FAD2.1 and FAD2.3) were digested using *BamHI* and *Alw26I* restriction enzymes (Fermentas) respectively, at 37 °C overnight. The restriction fragments length of PCR products were 224 and 17 bp, 130 and 110 bp for CC genotype of FAD2.1 SNP and FAD2.3 SNP, respectively. All digested products were separated by electrophoresis on 3% Nusieve ethidium bromide-stained agarose gels and visualized under UV light.

### Olive oil extraction

The olive oil samples were obtained from fully ripened olives coming from various dual purpose and table Tunisian olive cultivars. After harvesting, the olive fruit samples were immediately transported to the laboratory. Olive oil is produced by grinding 2.5 Kg stoned olives and extracted by a mechanical means. The procedure for monovarietal oil production followed the standard methods used in oil factories, including milling, malaxation for 30 min at 25 °C, centrifugation at 2000 g for 3 min and olive oil was obtained by natural decantation. Samples were stored into dark glass bottles at 4 °C until fatty acids composition analysis.

### Fatty acids composition

The fatty acid methyl esters (FAMEs) were prepared as described by European Union standard methods (Commission Regulation (EEC) no. 2568/91). FAMEs were prepared by vigorously shaking a solution of oil in hexane (0.2 g in 3 mL) with 0.4 mL of 2 N methanolic potassium hydroxide, and analysed by gas chromatography with a Shimadzu chromatograph equipped with a flame ionization detector (FID), and a fused silica column (30 m length × 0.32 mm i.d. and thickness, 0.25 μm, formed with 50% cyanopropylmethyl- 50% phenylmethyl-polysiloxane). An injection volume of 1 μl was used. The carrier gas was nitrogen with a flow rate of 1 mL/min. The injector and detector temperatures were set at 220 °C, whereas the oven temperature was held at 180 °C. Six fatty acids including C_16:0_, C_16:1_, C_18:0_, C_18:1_, C_18:2_, C_18:3_ were identified from their retention times.

### Statistical analysis

The analysis of the relationship between FAD2.1 and FAD2.3 SNP markers and the fatty acids composition was performed in many steps using several statistical techniques.

For Fatty acids composition, the t- test or one-way analysis of variance (one-way ANOVA) was used to assess the significant difference between the means of genotype groups for each SNP. To examine the association of the two SNPs simultaneously with fatty acids composition, a variance multi-way analysis (only main effects are included) was performed. Binary logistic regression was also used to test the associations of the two SNPs with fatty acids composition separately.

The Pearson’s correlation analysis was used to test associations between variables. All analyses were performed using R program. Two-sided *P*-values < 0.05 were considered statistically significant. Moreover, R language was used to draw the Directed Acyclic Graph (DAG), using the ‘growshrink’ algorithm. The algorithm efficiently filters links out of a full skeletal DAG, in which all nodes are primarily connected (except those having no relationships with others), based on tests of conditional independence between a pair of nodes given all possible subsets of the rest. Logical rules are applied to establish the direction of links (conditional dependence between variables), so that cycles are not introduced and patterns of conditional independence found in the data match the generated DAG. We estimated link influence in the final DAG by calculating the regression beta-coefficient for each potential causal effect in which the variable at the base of the arrow (‘cause’) was considered a covariate, and the variable at the head of the arrow (‘effect’) was considered the outcome or dependent variable. The advantage of Bayesian network is to deduct all parent nodes which are directly dependent on child nodes.

## Results

### Characteristics of the studied SNP markers

In the present study, PIC values observed in olive cultivars for FAD2.1 and FAD2.3 markers were 0.469 and 0.496, respectively (Table 1). The FAD2.3 SNP marker is more polymorphic than the FAD2.1 marker. In fact, this result demonstrates that FAD2.3 is slightly more informative than FAD2.1 marker and it is able to distinguish between studied olive oils. This result is consistent with the finding of Ben Ayed et al. [8], who demonstrated a highest discriminating power of FAD2.3 to distinguish between olive tree cultivars. Similar values were found by Reale et al. [9] in DNA samples extracted from 65 Italian olive cultivars.

**Table 1:** SNP marker panel information

Gene name	GenBank Accession Number	SNP code	Tm^a^	PIC^b^
*Fatty acid desaturase*	AY083163	FAD2.1(T/C)	57	0,469
		FAD2.3(C/G)		0.496

^a^annealing temperature for PCR amplification

^b^for each locus the polymorphism content information (PIC)

The allele frequencies of the two studied SNPs showed dominance of the heterozygous genotypes as well as for FAD2.1 and FAD2.3 markers (the frequency is about 75%).

### Genotypic associations of FAD2.1 and FAD2.3 SNPs with fatty acids composition by using bivariate and multivariate statistical analyses:

It has been reported that *FADS2* gene was implicated in the transformation of the monounsaturated oleic fatty acid (C18:1) to the polyunsaturated linoleic fatty acid (C18:2), therefore, we analyzed the association of FAD2.1 (C/T) and FAD2.3 (C/G) polymorphisms and the fatty acids composition of each olive oil sample.

### Correlation between FAD2.1 SNP polymorphisms and fatty acid compositions

For FAD2.1 marker analysis, we found only two genotypes for this SNP: CT and TT. About 75% of the varieties were heterozygous CT-FAD2.1. Table 2 shows results of *p*-values generated by t-test. Highly significant associations of this marker with the accumulation of the fatty acids are proved. In fact, three highly associations were established with the accumulation of the oleic monounsaturated fatty acid (C18:1; *p = 0.003*), linoleic polyunsaturated fatty acid (C18:2; *p = 0.012*), linolenic polyunsaturated fatty acid, (C18:3; *p = 0.006*) and three associations with two saturated fatty acids (C16:0; *p = 0.024*, and C18:0; *p = 0.041*).

**Table 2:** The association between FAD2.1 SNP and Fatty acids composition

Parameters		C16:0		C16:1		C18:0		C18:1		C18:2		C18:3	
SNPs		Mean ± SD	*P*	Mean ± SD	*P*	Mean ± SD	*P*	Mean ± SD	*P*	Mean ± SD	*P*	Mean ± SD	*P*
FAD 2.1	CT	13.778 ± 2.45	***0.024***	1.348 ± 0.73	*0.357*	2.14 ± 0.316	***0.041***	69.8 ± 3.85	***0.003***	11.62 ± 3.01	***0.012***	*0.59* ± 0.1	***0.006***
	TT	17.767 ± 1.15		1.82 ± 0.74		1.66 ± 0.27		59.63 ± 4.05		18 ± 3.6		*0.83* ± 0.115	

Bold values: each variable that has statistical significance for all tests was declared when *P*-values of t-test are <0.05; ***P***: *P*-value of student test; **SD**: standard deviation

These associations are confirmed by multivariate analysis, in fact, Table 3 shows that high significant associations were found between FAD2.1 SNP and four parameters which are C16:0, C18:1, C18:2 and C18:3. However, a significant difference of the average rate in C18:1 between the heterozygote varieties with CT-FAD2.1 genotype and TT-CALC genotype (*p = 0.003*) was observed. A positive relationship between the rate of linoleic fatty acid (C18:2) and the genotype variation for this marker (*p = 0.012*) can also be noted. In this case, the varieties with CT genotypes have the lowest rate of C18:2 and the highest level of C18:1. These heterozygous genotypes concern essentially Zarrazi and Chetoui cultivars. The third significant association was shown with the linolenic fatty acid (*p = 0.006*). In fact, the homozygous varieties TT have higher level of C18:3 and lower rate of C18:1 than the heterozygous varieties CT.

**Table 3:** The association between FAD2.1 and FAD2.3 SNPs and Fatty acids composition

	C16:0	C16:1	C18:0	C18:1	C18:2	C18:3
FAD2.1	0.166*	0.677*	0.058*	0.197*	0.478*	0.034*
	0.026**	0.312**	0.039**	0.007**	0.016**	0.11**
FAD2.3	0.851*	0.989*	0.577*	0.345*	0.328*	0.774*
	--	--	--	--	--	--

*P-values given by the variance analysis; **P-values given by the binary logistic regression analysis; Bold values: each variable that has statistical significance for all tests was declared when *P-values* are <0.05

### Correlation between FAD2.3 SNP polymorphisms and fatty acid compositions

Concerning the second SNP (FAD2.3), two highly significant associations of this marker with two parameters are proved. As shown in Table 3, correlations are observed with the oleic acid (*p = 0.003*) and the linoleic acid (*p = 0.004*). The homozygous varieties GG-FAD2.3 (Chemlali Sfax and Meski cultivars) have a lower oleic acid level than the heterozygous varieties CG (other cultivars) and the homozygous variety CC (Chemchali). However, the rate of C18:1 was significantly higher in the varieties carrying the heterozygous genotype CG-FAD2.3 (especially the two dual purpose cultivars: Oueslati (C18:1 rate = 74.5) and Zarrazi (C18:1 rate = 74.6) and the table olive cultivar: Tounsi (C18:1 rate = 74.8)) than the homozygous genotypes (GG). Moreover, CC varieties have a medium oleic fatty acid rate than the heterozygous genotypes CG.

### Genotypic associations of FAD2.1 and FAD2.3 SNPs with fatty acids composition by Bayesian networks modeling:

Bayesian networks (BNs) are directed acyclic graphs composed by nodes (variables of the problem) and arcs that encode conditional probabilistic independencies between the nodes. These graphical models are able to explain probabilistic interactions connecting variables. In fact, they have proven to capture causal relationships between variables and they can show excellent forecast accuracy even with relatively small sample data sizes [10]. To achieve the mentioned objectives, a Bayesian network modeling was used.

**Fig. 1:**
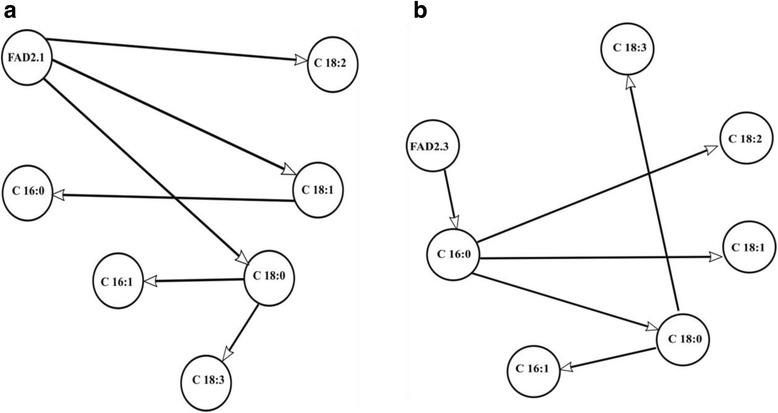
Directed acyclic graph representing possible fatty acid parameter connexions with the first FAD2.1 SNP (**a**) and the second FAD2.3 SNP (**b**)

Firstly, we considered 7 nodes as represented in Fig. 1a. Correlation coefficients among fatty acid compositions in olive oil cultivars are presented in Table 4. Indeed, the oleic acid and the linoleic acid amounts are the most important nutritional properties in olive oil [11]. In our study, base of core “FAD2.1” has three connections: “FAD2.1” is negatively related with oleic mono-unsaturated acid “C18:1” and on the other hand, positively correlated with linoleic poly-unsaturated acid “C18:2” and linolenic poly-unsaturated acid “C18:3”.

**Table 4:** Pearson’s correlations of FAD2.1 and FAD2.3 markers with fatty acids composition of the studied olive oil cultivars

Parameters	FAD2.1		FAD2.3	
	*r*	*p*	*r*	*p*
C16.0	0.643	***0.024***	0.503	0.095
C16.1	0.292	0.357	0.257	0.42
C18.0	−0.596	0.041	−0.29	0.36
C18.1	−0.778	***0.003***	−0.781	***0.003***
C18.2	0.693	***0.012***	0.759	***0.004***
C18.3	0.735	***0.006***	0.465	0.127

Bold and italic values: each variable that has statistical significance for all tests was declared when *P*-values are < 0.05

Additionally, Fig. 1b. shows that oleic acid “C18:1” was positively influenced by the saturated stearic acid “C18:0” and negatively influenced by the saturated palmitic acid “C16:0”. The latter is negatively influenced by “C18:1” and positively influenced by “C18:2”. Besides, C16:0 is directly influenced by the “FAD2.3 marker”. FAD2.1 and FAD2.3 markers play a key role in the fatty acids composition of each olive oil cultivars. This finding could be explained by the fact that FAD2.1 SNP and FAD2.3 SNP are located in the *FADS2* gene which is involved in the process of synthesis of the unsaturated fatty acid [6], suggesting the direct effect of the FAD2.1 and FAD2.3 genotype variations on the percentage of poly-unsaturated fatty acid (as C18:2 and C18:3) for each variety.

## Discussion

In recent years, several studies showed that the consumption of olive oil was correlated with the prevention and therapy for many diseases. In fact, olive oil contains many bioactive molecules that could explain its multiple therapeutic effects towards many diseases including cardiovascular pathologies, cancer, arthrosclerosis, osteoporosis and immunity deregulation. Among these biomolecules: mono-unsaturated free fatty acid, particularly oleic acid is the most important component [12–14]. However, its amount depends principally on the cultivar. Thereafter; the benefit of olive oil is directly related to the olive cultivar and the olive oil chemical composition. However, little is known about the ultimate association genotype and oleic acid variation between olive cultivars. Our current findings, demonstrated that oleic acid amount is stringently related to the presence of two SNP markers localized in the *FADS2* gene, involved in the process of synthesis of the unsaturated fatty acid, particularly in the desaturation of the oleic acid (C18:1) to the linoleic acid (C18:2).

In the present study, after genotyping these two SNPs (FAD2.1 and FAD2.3), we determined the fatty acids composition of each studied olive oil cultivar. Then, in order to examine the variation effect of these two SNPs on the fatty acids profile, especially, oleic and linoleic fatty acid, statistical analysis based on bivariate and multivariate analyses of six fatty acid variables were applied. Subsequently, Pearson’s correlation and bayesian networks of fatty acid parameters and the two studied SNP were employed to confirm the obtained associations.

Our results showed that the oleic fatty acid (C18:1) and the linoleic fatty acid (C18:2) levels were related to FAD2.1 and FAD2.3 SNPs genotypes. These two SNPs were significantly associated with C18:1 and C18:2 proportions in the olive oil cultivars. We also showed an association between these markers and saturated fatty acids, particularly, C16:0. These results suggested that these two loci may be an oleic and linoleic fatty acid –specific SNPs.

Accordingly to the first SNP (FAD2.1), its correlation with the level of C18:1 and C18:2 was proved with the statistical and modeling analyses used in this study. In fact, we showed that the homozygous genotype TT was positively correlated with the level of C18:1and negatively correlated with the level of C18:2. Furthermore, we demonstrated that the FAD2.3 SNP genotype variations were significantly associated with the fatty acids levels. In fact, the homozygous FAD2.3-GG genotype was negatively correlated with the C18:1 level (*r* = −0.781, *p* = 0.003) and positively correlated with C18:2 level (*r* = 0.759, *P* = 0.004). This results concern essentially two cultivars: Chemlali Sfax and Meski, which had the haplotype TT-FAD2.1-GG-FAD2.3.

Oleic and linoleic fatty acid proportion differences in olive oil cultivars are well-established, indeed, several previous studies [15, 16] explained that the fatty acid content of the olive oil was influenced by the variety, the geographical location, the climate and the technological process used in oil extraction. Nevertheless, until now, no work studied nor demonstrated the genetic starting point of these fatty acid variations. Thus, our findings indicated that the two SNP FAD2.1 and FAD2.3 might be a useful tool to elucidate the genetic basis of fatty acid variations in olive oil cultivars and it might be a predictive haplotype marker to identify high quality of olive oil; with beneficial effect on health and especially in hypertension and cardiovascular prevention diseases. Besides, mutations in these two SNPs appeared to cause different fatty acid compositions among olive oil cultivars, suggesting that the cultivar fatty acid composition differences reflect genetic diversity, which may involve differences in gene expression and function. It should be noted that the fatty acid difference in olive oil cultivars involve many genetic and environmental factors and their complex interactions. Our finding might partially explain this variability. The choice of the best varieties of virgin olive oil based on its richness in monounsaturated oleic fatty acid is necessary for use as a traditional nutraceutical food. Nutraceuticals can have a fundamental role and can be adopted in preventing or/and treating dyslipidaemia, especially when considering patients who are intolerant to hypolipidemic medication, suffering from severe lipid disorders or whose medication treatment is not able to obtain good results. Nevertheless, until now the question about the lipid-lowering properties of virgin olive oil is complex. The process underlying this property is still under debate. Estruch et al. [17] studied the impact of the use of virgin olive oil as a supplement to the Mediterranean diet and they concluded that these nutraceuticals can reduce incidence of major cardiovascular events induced by dyslipidaemia. The exact inner mechanisms underlying such action are not fully understand but may be related to numerous hypotheses. In fact, nutraceuticals actions of functional food ingredients (for example virgin olive oil) in lipid mechanism in human body could be explained by several biochemical pathways able to influence lipid disorder in cell. The recent work from Scicchitano et al. [18] explicated that the nutraceuticals may represent useful compounds in the management of lipid disorders. In fact, it could be related to reducing 7α-hydrolase, deceasing 3-hydroxy-3-methyl glutanyl-CoA reductase mRNA levels or reducing the secretion of very low-density lipoprotein.

A major strength of our study is being the first demonstrating the impact of fatty acid related SNPs on variability levels of fatty acid content in virgin olive oil studied cultivars. Although the influence of environmental conditions in fatty acid rates has been studied previously [19]. However, no study until now revealed the genetic basis of the fatty acid fluctuations. Thus, we discussed in our present research the impact of fatty acid composition differences on the association of FAD2.1 and FAD2.3 SNPs with saturated, mono and polyunsaturated fatty acid profiles among on virgin olive oil cultivars. Besides, we focused on the effect of two SNPs and we did explore interaction effects between them. Indeed, the studied virgin olive oil samples used in this work were taken from Tunisia and might not be representative of entire olive population. However, since molecular markers, including SNP markers, statistical and modelling analyses were used, which are effective and reliable tools, it may be considered that this work may be representative to study the authenticity of olive oil in the world based on correlation of these SNP markers with the fatty acid profile. Further large and internationally representative olive oil samples and analyses are necessary to confirm our findings.

## Conclusions

Our results establish and demonstrate, to the best of our knowledge for the first time, that oleic acid, the main monounsaturated fatty acid level of olive oil, is associated with two SNPs (FAD2.1 and FAD2.3) located in the coding region of *FADS2* gene. These associations differed between studied cultivars, which might explain that the cultivar fatty acid composition differences reflect genetic diversity, including variability in gene regulation activity and metabolite pathways. Therefore, these two SNP markers could be informative about the characteristics and quality of olive oils and thereafter could recommend the best olive oil cultivars for consumers according to their fatty acid compositions and SNP haplotype.

## Abbreviations

*FADS2*: *Fatty acid desaturase 2*
SNP: Single nucleotide polymorphism
